# Purification, Preliminary Structure and Antitumor Activity of Exopolysaccharide Produced by *Streptococcus thermophilus* CH9

**DOI:** 10.3390/molecules23112898

**Published:** 2018-11-06

**Authors:** Naxin Sun, Huiping Liu, Shaojuan Liu, Xinyuan Zhang, Pei Chen, Weihong Li, Xiangxin Xu, Wentan Tian

**Affiliations:** Key Laboratory of Food Nutrition and Safety, Ministry of Education, Department of Food Engineering and Biotechnology, Tianjin University of Science & Technology, Tianjin 300457, China; snxtust@sina.com (N.S.); liu_fendouzheye@126.com (S.L.); zhangxinyuan628540@163.com (X.Z.); chenpei689689@163.com (P.C.); fallinlove7384@163.com (W.L.); 17808022@mail.tust.edu.cn (X.X.); tianwentan@mail.tust.edu.cn (W.T.)

**Keywords:** *S. thermophilus* CH9, exopolysaccharide, preliminary structure, antitumor activity

## Abstract

In the present study, the preliminary structure and in vitro antitumor activity of three exopolysaccharides (EPSs) from *Streptococcus thermophilus* CH9 were investigated. Then, three purified fractions of EPS-1a, EPS-2a, and EPS-3a were obtained by chromatography using DEAE-52 cellulose and Sephadex G-100, respectively. The average molecular weight of EPS-1a, EPS-2a, and EPS-3a, were 1.80 × 10^6^, 1.06 × 10^6^ and 1.05 × 10^6^. The monosaccharide composition of EPS-3a was dramatically different from the others. The EPS-1a and EPS-2a were mainly composed of mannose, in a ratio of 69.82% and 57.09%, respectively, while EPS-3a was mainly composed of glucose (63.93%), without mannose. In addition, the surface morphology observed suggested that there were protein particles on the sugar chain of EPS-3a and EPS-3a was a protein-containing polysaccharide. Furthermore, EPS-3a exhibited higher antitumor activity against human liver cancer HepG2 cells in vitro. The antitumor activity of EPS-3a in HepG2 cells was associated with cell apoptosis. HE staining and Hoechst 33342 staining showed that with the treatment of EPS-3a, HepG2 cells had typical morphological changes. Flow cytometry analysis showed that the cell cycle was arrested at G0/G1 phase.

## 1. Introduction

In recent years, microbial polysaccharides led increased attention in the fermented dairy industry, not only because of its viscous properties [1], but also because it is an important source of natural alternatives to commercial additives of plant or animal origin of safe status. Microorganisms can secrete polysaccharide layers on their cell surface which, together with a few glycoproteins, are grouped within the general term “glycocalyx”. These exocellular polymers comprise the capsular polysaccharides, which form a cohesive layer or capsule covalently linked to the cell surface. The long-chain polysaccharides contain branches and some repeating units of sugars or sugar derivatives, such as glucose, fructose, mannose, and galactose etc., which are related to their particular biological activity [2].

Among the wide variety of EPS-producing bacteria, lactic acid bacteria (LAB) have gained special attention, due to the remarkable property of the polymers they synthesize, which have beneficial effects on human health, such as cholesterol-lowering ability [3], antitumor [4,5], immunostimulatory [6], antibiofilm [7], and antioxidant activity [8]. The EPS produced by *Lactobacillus helveticus* MB2-1 had strong scavenging on free radicals and chelating activities on ferrous ions [9]. The EPS produced by *Lactobacillus plantarum* YW32 had strong scavenging capacities on hydroxyl and superoxide free radicals, concentration-dependent inhibition on biofilm formation of several pathogenic bacteria, and good inhibition activity on HT-29 cells of colon cancer [10]. The EPS obtained from *Lactobacillus confusus* TISTR 1498 partially hydrolyzed EPS-stimulated RAW264.7 cells through the activation of NF-κB and JNK pathways [11]. Therefore, lactobacillus extracellular polysaccharides can be used as a natural antioxidant, antimicrobial agent, antitumor, and immune-enhancing drug in the pharmaceutical field. Meanwhile, their good water-retaining and rheological properties can also accelerate their development in the food industry. The purified EPS obtained from *Streptococcus thermophilus* ST-143 was added to milk, and it was found that the gel stiffness and viscosity of the fermented milk increased with the increase of EPS [12]. The addition of lactobacillus EPS could promote the absorption of water and prolong the shelf life of bread [13]. Several authors have reported that *S. thermophilus*, classified as GRAS (generally recognized as safe), has the ability to produce a wide variety of EPSs [14]. For this reason, many studies on preliminary structural of EPS produced by *S. thermophilus*, with the help of advanced analytical techniques, have been performed.

WTO reported that more than 7 million people died of cancer, a proliferation disorder involving an obstruction to apoptosis, and its continued spread with increasing incidence had continued into the 21st century. Microbial secondary metabolites, including EPSs from microorganisms, represent a large source of compounds endowed with ingenious structures and potent biological activities. It has been reported that EPSs, natural polymers which have a complex chain structure, have antitumor activity [15]. Therefore, more attention has been paid to microbial polysaccharides.

In this study, an attempt was made to characterize the chemical and physical properties of EPSs isolated from *S. thermophilus* CH9. Their antitumor activities were tested on human liver cancer HepG2 cells. The physicochemical characteristics of the EPSs were analyzed based on their monosaccharide composition and physical properties. The EPSs were found to induce cytotoxicity, cell cycle arrest, and apoptosis in HepG2 cells.

## 2. Results and Discussion

### 2.1. Isolation and Purification of EPS

**Figure 1:**
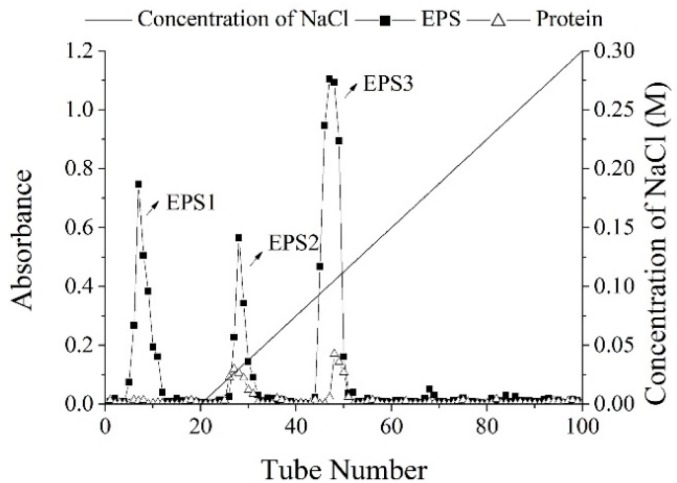
The elution curve of crude exopolysaccharide (EPS) on DEAE-52. The carbohydrates were detected via UV absorption at 490 nm, and proteins were detected at 280 nm. The eluent solvents were distilled water and a linear gradient of NaCl from 0 to 0.3 M.

**Figure 2:**
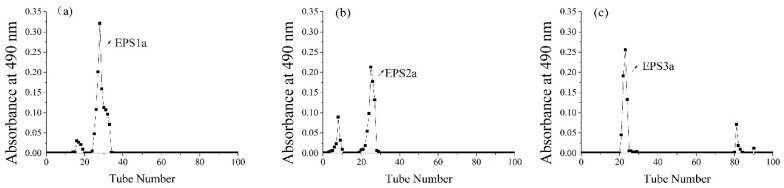
The elution curve of EPS1 (**a**), EPS2 (**b**), and EPS3 (**c**) on G-100. The carbohydrates were detected via UV absorption at 490 nm. The eluent solvent was distilled water.

The sugar content of EPS crude polysaccharide was 87.33%, and its yield was 219.25 mg/L. In Figure 1, the solution of crude EPS was applied to a column of DEAE-52, resulting in three fractions, which eluted with the distilled water, 0.05 M, and 0.1 M NaCl solutions, named as EPS-1, EPS-2, and EPS-3 (the ratio was 31.32%, 24.33%, and 44.35%), respectively. Since DEAE is an anion exchanger with positive charge, in the process of balance, it could bind to negatively charged balance ions in the buffer [16]. After loading the EPS crude polysaccharide, the acidic polysaccharides with negative charge could exchange with balance ions, and bind to the chromatographic column. When eluting, the neutral groups were removed with distilled water, and the negatively charged groups were removed with NaCl. The EPS1 was eluted by distilled water, indicating that EPS1 was a neutral polysaccharide. However, EPS2 and EPS3 were eluted by NaCl, indicating that they were ascribed to acidic polysaccharides. In Figure 2, three independent fractions (EPS-1a, EPS-2a, and EPS-3a) were obtained by a column of G-100. Then, their carbohydrate and protein contents were detected, indicating that EPS-3a contained the highest content of protein.

### 2.2. Molecular Weight

**Figure 3:**
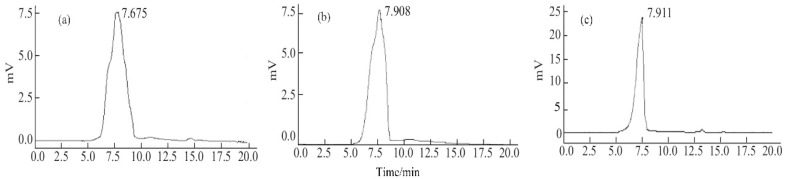
The HPGPC profiles of EPS1a (**a**), EPS2a (**b**), and EPS3a (**c**) determined by a HPLC System. The mobile phase was ultrapure water at a flow rate of 0.8 mL/min. The temperature of the chromatographic column (Shodex OHpak SB-804 HQ) was 30 °C.

The peak of EPS-1a, EPS-2a, and EPS-3a showed a single symmetric peak (Figure 3). The average molecular weight of EPS-1a, EPS-2a, and EPS-3a were 1.80 × 10^6^, 1.06 × 10^6^ and 1.05 × 10^6^.

### 2.3. Monosaccharide Composition Analysis

**Figure 4:**
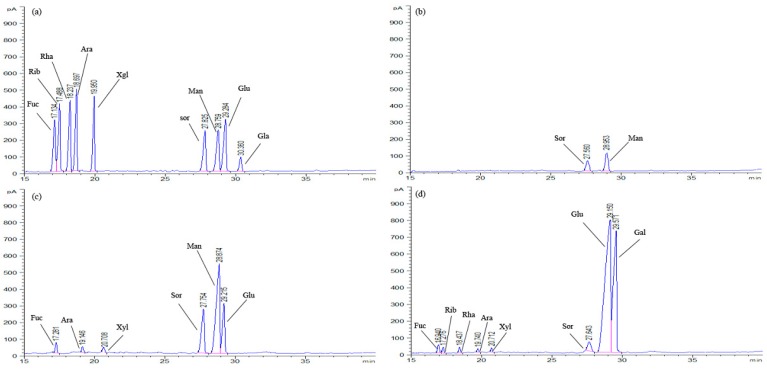
The GC profiles of standard monosaccharides (**a**), EPS-1a (**b**), EPS-2a (**c**), and EPS-3a (**d**) determined by GC system.

The monosaccharide compositions of the three purified fractions (EPS-1a, EPS-2a, and EPS-3a) were analyzed using GC, and the data were presented in Figure 4 and Table 1. Notably, mannose was found to be the most abundant monosaccharide, in a ratio of 69.82% and 57.09%, respectively, for EPS-1a and EPS-2a. Meanwhile, EPS-3a was composed of fucose, ribose, rhamnose, arabinose, xylose, sorbose, glucose, and galactose, in a ratio of 1.07%, 0.63%, 0.59%, 0.35%, 0.41%, 2.03%, 63.93% and 36.07%. The presence of different sugar moieties indicated that EPSs were heteropolysaccharides. Vijayendra et al. (2008) reported the exopolysaccharide produced by a non-ropy strain of *Leuconostoc* sp. CFR 2181 was composed of glucose (91%), rhamnose, and arabinose (1.8% each) [17]. Marshall et al. (1995) reported the production of a phosphopolysaccharide by *Lactococcus lactis* subsp. *cremoris* LC330, which consisted of glucose, rhamnose, galactose, and glucosamine in an approximate ratio of 6:5:4:1, respectively [18]. Interestingly, ribose, rhamnose, and galactose were detected only in EPS-3a. Besides, the result indicated that glucose was the main sugar constituent of EPS-3a and, probably, the primary hydroxyl groups in glucose were oxidized to form acidic groups, which accounted for the acidic property of EPS-3a.

### 2.4. SEM Analysis

**Figure 5:**
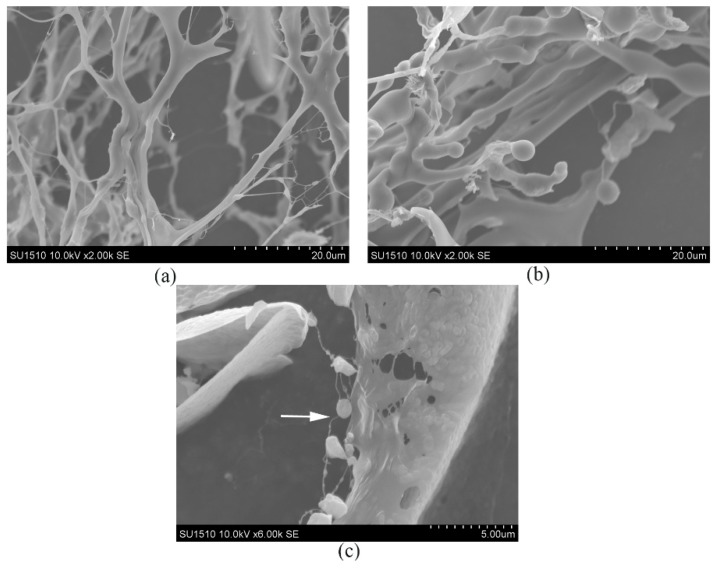
The microstructure and surface morphology micrographs of EPS-1a (**a**), EPS-2a (**b**), and EPS-3a (**c**) observed by SEM at an accelerating voltage of 10 kV.

In an attempt to study microstructural difference in the three fractions, scanning electron microscopy was used to depict the surface morphology of macromolecules. SEM image (shown in Figure 5a) indicated that EPS-1a had a random, web-like and highly branched strcuture [19]. Interestingly, the strcuture of EPS-2a was significantly different from EPS-1a. The image of EPS-2a (shown in Figure 5b) revealed a smooth, compact and cluster-like structure [20]. In Figure 5c, the EPS-3a exhibited a smooth and porous structure. Besides, there were bead-like substances adhered to polymeric chains. It was supposed that the bead-like substances might be protein particles and EPS-3a might be a protein-containing polysaccharide.

### 2.5. AFM Analysis

The EPS-1a was spherical structure, and the height and diameter of spheroids were 0.109–2.85 nm and 0.039–0.072 nm, respectively. Generally, the height of a single polysaccharide chain was 0.1–1 nm, while the height of EPS-1a was higher than that of a single polysaccharide chain, thus we speculated that there were inter- and/or intra-molecular aggregation [21].

**Figure 6:**
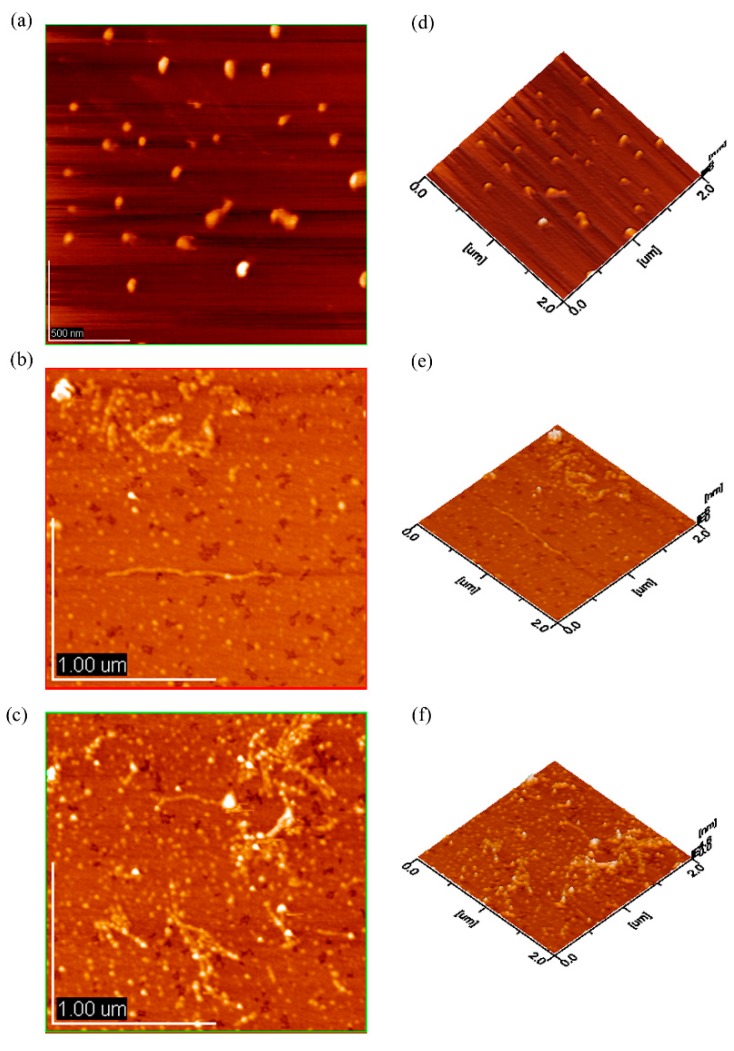
Atomic force microscopy (AFM) 2-D ((**a**), (**b**), and (**c**)) and 3-D ((**d**), (**e**), and (**f**)) images of molecular structure of EPS-1a (**a**,**d**), EPS-2a (**b**,**e**), and EPS-3a (**c**,**f**). The concentration was 100 μg/mL.

Meanwhile, the topographical AFM images of EPS-2a were round lumps and chains, which could be seen in Figure 6b,e. The maximal height of lumps was 1.23 nm, but the lumps and chains formed an irregular reticulation shape. A similar experiment reported about an acidic polysaccharide from a mycelial culture of *Cordyceps sinensis* fungus Cs-HK1 [22].

The AFM image of EPS-3a was dramatically different from the others (Figure 6c,f). The worm-like strands connecting the network might be formed by aggregation of the polysaccharide chains and branches, and the lightspots that existed in the chains presumably were the sites for connection to each polysaccharide molecule. A similar experiment was reported about an acidic polysaccharide from *Mesona* blumes gum [23].

### 2.6. Antitumor Activity against HepG2 Cells In Vitro

#### 2.6.1. Effect of EPS on HepG2 Proliferation

**Figure 7:**
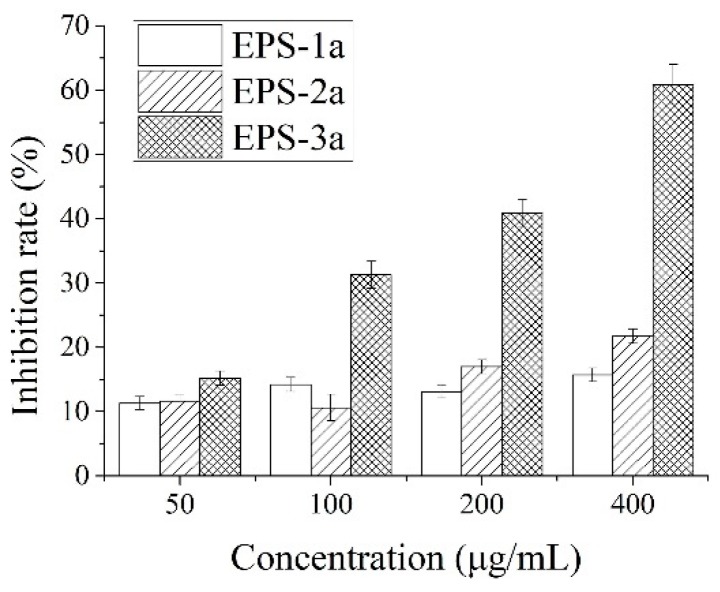
Test of EPS on cell proliferation of human liver cancer HepG2 cell line. The concentrations of EPS-1a, EPS-2a, and EPS-3a were either 50, 100, 200, and 400 μg/mL.

The effects on proliferation of the HepG2 cells were first investigated in order to evaluate the potential antitumor activity of EPS-1a, EPS-2a, and EPS-3a. As shown in Figure 7, various concentrations (50, 100, 200, and 400 μg/mL) of EPS, at the time of 24 h, caused the proliferation inhibition rate of HepG2 cells to increase in a dose-dependent manner. EPS-3a exhibited the strongest inhibition activity in comparison to EPS-1a and EPS-2a. The IC50 (cell inhibition ratio was nearly 50%) of EPS-3a was 313.75 μg/mL, while the inhibition ratio of EPS-1a and EPS-2a were no more than 50%, regardless of the changes of dose.

Recently, studies related to the antitumor activities of polysaccharides have increased. It has been demonstrated that their antitumor effects depend on the molecular weight, chemical composition, structure of the polymeric backbone, degree of branching, and so on [24]. In our study, EPS-3a showed the highest in vitro antitumor activity, more so than the other two fractions. The differences of antitumor activity for EPS-1a, EPS-2a, and EPS-3a might be due to their differences in monosaccharide composition and contents of protein. Besides, the antitumor activity was deeply dependent, among other factors, on the three-dimensional structure and conformation of the polymeric chains. Comparing with EPS-1a and EPS-2a, EPS-3a contained a higher ratio of glucose (63.93%) in monosaccharide composition, and relatively higher contents of protein. Apart from this, the reason that EPS-1a exhibited relatively lower antitumor activity might be due to its origin (the fraction eluted with water from the column of DEAE-52 cellulose during the purification of crude EPS) and monosaccharide composition (sorbose 30.18% and mannose 69.82%). It was reported that polysaccharides with high antitumor activity from the fruiting bodies of mushrooms were mostly heteropolysaccharides consisting of galactose, glucose, mannose, and fucose and, also, those from the mycelia of mushrooms are mainly protein-containing glucans [25]. Incidentally, EPS-3a was also a protein-containing heteropolysaccharide.

#### 2.6.2. HE Staining

**Figure 8:**
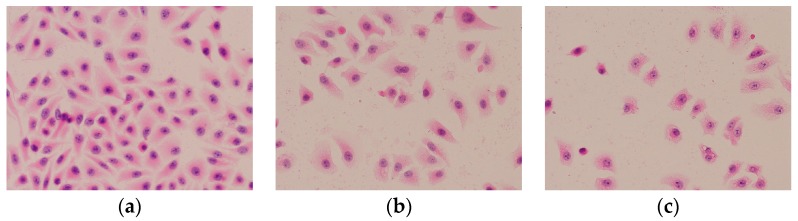
Effect of EPS-3a induced HepG2 cells apoptosis by HE staining (40×). Cells were treated with EPS-3a for different time (24 h, 48 h and 72 h). The concentration of EPS-3a was 313.75 μg/mL. (**a**) Control, 0 h. (**b**) Cells treated with EPS-3a for 24 h. (**c**) Cells treated with EPS-3a for 48 h.

Apoptosis refers to the morphological features such as cytoplasmic blistering, chromatin condensation, nuclear fragmentation, cell roundness, and cell contraction [26,27]. To confirm whether the loss of cell viability was due to apoptosis or not, cell changes in morphology were evaluated by HE staining. As shown in Figure 8, after treatment with EPS-3a, marked morphologic changes in cell nuclei were observed, compared with the control.

As shown in Figure 8a, the control cells grown in a good state appeared in a spindle or triangle irregular shape, and the nucleus and cytoplasm were clearly distinguishable. When treated with EPS-3a for 24 h (Figure 8b), it was found that some cells began to become round and shed out, but the cells were intact, and some nuclei showed agglutination. When the treatment time increased to 48 h (Figure 8c), the cells showed apoptosis morphological changes: cytoplasm concentration, partial nucleus concentration, and nuclear disintegration.

#### 2.6.3. Hoechst 33342 Staining

**Figure 9:**
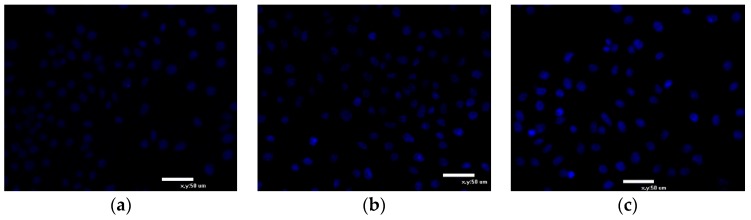
Effect of EPS-3a induced HepG2 cells apoptosis by Hoechst 33342 staining. Cells were treated with EPS-3a for different time (24 h, 48 h and 72 h). The concentration of EPS-3a was 313.75 μg/mL. (**a**) Control, 0 h. (**b**) Cells treated with EPS-3a for 24 h. (**c**) Cells treated with EPS-3a for 48 h.

Apoptosis was further confirmed by analyzing the nuclear by Hoechst 33342 staining. The fluorescent dye, Hoechst 33342, is widely used to stain DNA for evaluating apoptosis of viable cells. It is a blue fluorescent dye that can penetrate cell membranes and is less toxic to cells [28,29]. A small amount of Hoechst 33342 can enter normal cell membranes, so normal cells are light blue. When apoptosis occurs, the permeability of cell membrane enhances, DNA structure changes, so the dye Hoechst 33342 can bind to DNA more effectively and displays bright blue fluorescence. Cell apoptosis can be judged by blue fluorescence level. In the control group (Figure 9a), nuclei were round and slightly stained. After being incubated with EPS-3a for 24 h (Figure 9b), some nuclei appeared hypercondensed (brightly stained). After treatment for 48 h, nuclei stained by Hoechst 33342 increased significantly and the chromatin further condensed (Figure 9c). The results demonstrated that EPS-3a could induce apoptosis of HepG2 cells.

#### 2.6.4. Cell Cycle Analysis

**Figure 10:**
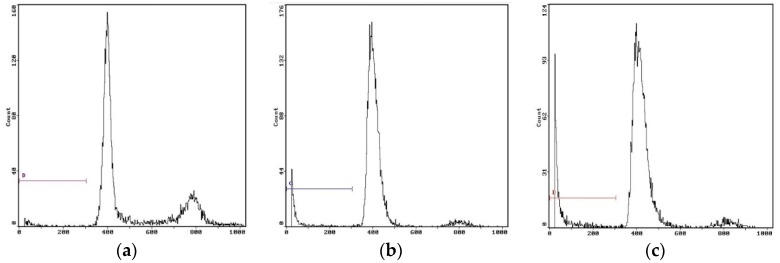
Flow cytometric analysis of DNA fragmentation. (**a**) Control, 0 h. (**b**) Cells treated with EPS-3a for 24 h. (**c**) Cells treated with EPS-3a for 48 h.

Flow cytometry is a useful tool to examine whether EPS-3a could interfere with the cell cycle of HepG2 cells stained with propidium iodide. Many antitumor agents and DNA-damaging agents arrest the cell cycle and, then, induce apoptotic cell death [30]. HepG2 cells in the control showed normal cell characteristics: the ratio of apoptotic cells was 2.59%, at the same time, 63.70% of the cells were in G0/G1 phase (Figure 10a and Table 2). After incubation with EPS-3a for 24 h, 7.45% of cells had undergone apoptosis and the cell proportion in G0/G1 phase was 75.81% (Figure 10b and Table 2). After 48 h, the apoptotic ratio increased to 14.70%, and the percentage of cells in G0/G1 phase was 89.70% (Figure 10c and Table 2). These results indicated EPS-3a could induce HepG2 cell apoptosis, and the cell cycle was arrested at G0/G1 phase. All phenomena suggested that EPS-3a could induce apoptosis in HepG2 cells [31].

## 3. Materials and Methods

### 3.1. Preparation and Purification of Polysaccharides

#### 3.1.1. Strain and Growth Medium

*S. thermophilus* CH9 isolated from curd was stored at −80 °C in MRS medium containing 25% (*v*/*v*) glycerol as cryoprotectant. The growth medium of *S. thermophilus* CH9 was skim milk-based medium containing 0.5% sucrose, 1.5% soy peptone, and 0.3% Tween 80.

#### 3.1.2. Preparation of Crude EPS

The crude EPS was prepared according to the reported method with some modifications [32]. *S. thermophilus* CH9 was cultured at 40 °C for 24 h on modified skimmed milk-based medium. Proteins were precipitated using trichloroacetic acid after adding neutral protease at 50 °C for 4 h, and then bacterial cells and precipitates were removed by centrifugation (10,000× *g*, 20 min, 4 °C). The supernatants were concentrated to one-fifth of their original volume with a rotary evaporator under reduced pressure. The resulting concentrate was mixed with three volume of absolute ethanol, stirred vigorously, and kept overnight at 4 °C, and then centrifuged (1000× *g*, 15 min). The precipitate was dissolved in distilled water, and insoluble material was removed by centrifugation. The partially purified EPS was obtained by dialysis (molecular weight cut-off 3500) against distilled water at 4 °C for 4 d, followed by lyophilization.

#### 3.1.3. Purification of Crude EPS

The crude EPS was purified continuatively by anion-exchange chromatography with DEAE-52 column (2.5 × 50 cm) and gel chromatography with Sephadex G-100 column (2.6 × 50 cm). In general, 8 mL of crude EPS solution (10 mg/mL) was applied to a column of DEAE-52, and the column was eluted with distilled water and a linear gradient NaCl, from 0 to 0.3 M, at a flow rate of 1mL/min. The eluates were collected by an automatic collector (3 mL/tube) and, then, the carbohydrate and protein contents were detected via UV absorption. The carbohydrate was determined by the phenol–sulfuric acid method, using glucose as the standard at 490 nm [33]. The protein content was determined by spectrophotometer at 280 nm. As a result, three fractions of polysaccharides (EPS-1, EPS-2, and EPS-3) were obtained, dialyzed (molecular weight cut-off 3,500) against distilled water, lyophilized, and further purified through a column of Sephadex G-100, producing EPS-1a, EPS-2a and EPS-3a, respectively. Finally, the three purified fractions were collected for further research, respectively.

### 3.2. Preliminary Structure of EPS

#### 3.2.1. Molecular Weight

The average molecular weight (M_w_) of the purified polysaccharides were determined by high-performance gel permeation chromatography (HPGPC) using a HPLC System (LC-20AT, Shimadzu Corp., Kyoto, Japan). The calibration curve was established with Dextran standards (670000; 410000; 150000; 80000; 50000). The standards used for the estimation of the M_w_ were dextrans. This meant that the obtained M_w_ values could be considered correct only in the case of flexible polymers. In all other case, the obtained M_w_ were to be considered just an estimation. Chromatographic column: Shodex OHpak SB-804 HQ (30 cm × 8 mm); mobile phase: ultrapure water; flow rate: 0.8 mL/min; column temperature: 30 °C; loading volume: 20 μL. The regression equation was as follows:

y = 13.125x^4^ − 491.54x^3^ + 6910.8x^2^ − 43243x + 101651 (1)

#### 3.2.2. Analysis of Monosaccharide Composition

The monosaccharide compositions of EPS-1a, EPS-2a, and EPS-3a were determined using the method reported by Gan et al. [34] with slight modification. The samples were hydrolyzed with 2 M trifluoroacetic acid (TFA) at 120 °C for 3 h. Then, the excess TFA was removed by evaporation at a temperature of 40 °C. The methanol was added into the dry sample, which was then acetylated by the addition of a mixture of pyridine and acetic anhydride. Finally, the acetylated samples were analyzed for identification and quantification of the monosaccharide by GC.

#### 3.2.3. Scanning Electron Microscopy (SEM) Analysis of EPS

The analysis of microstructure and surface morphology of the polymers was conducted by scanning electron microscopy (SEM, JEOL/EO, and model JSM-6380, JEOL Ltd, Tokyo, Japan) at an accelerating voltage of 10 kV. The conductive adhesive was cut to 0.5 cm with scissors, and pasted onto the sample table. Then, the EPS samples were taken with a cotton swab, and placed onto the conductive adhesive. The cotton swab was used to fully disperse the samples, and the unfixed samples were removed with a rubber suction bulb. Before SEM examination, all samples were coated with a layer of gold. After putting the sample table into SEM, the morphology of EPS was observed.

#### 3.2.4. Atomic Force Microscope (AFM) of EPS

The EPS-1a, EPS-2a, and EPS-3a were dissolved in distilled water with constant stirring (magnetically) overnight, respectively, so that the EPSs were dissolved completely. The sample solution was diluted with water to 100 μg/mL, and dropped on a surface of mica disk, and subsequently allowed to dry at room temperature. Finally, the AFM images were generated by atomic force microscope (JEOL JSPM-5200, JEOL Ltd, Tokyo, Japan) in tapping mode and three-dimensional images were obtained by SPM-offline 2.2 software (Shimadzu Corp., Kyoto, Japan). The driven amplitude was 0.430 V, and the cantilever oscillated at its proper frequency (158 kHz).

### 3.3. Assay of Antitumor Activity against HepG2 Cell Proliferation In Vitro

#### 3.3.1. Cell Lines and Culture, and MTT Assay

The human liver cancer HepG2 cells were obtained from Tianjin Medical University. The cells were grown in DMEM (high glucose) medium containing 10% (*v*/*v*) heat-inactivated fetal bovine serum, 100 units/mL penicillin, and 100 μg/mL streptomycin in a humidified incubator of 95% air and 5% CO_2_ at 37 °C.

The cell was measured by the 3-(4,5-dimethylthiazol-2-yl)-2,5-diphenyltetrazolium bromide (MTT) method. Firstly, the cells were plated on 96-well plate, with each well containing 1 × 10^4^ cells, and incubated for 24 h at 37 °C in a CO_2_ incubator. Then various concentrations of EPS (50, 100, 200, and 400 μg/mL) were added. When incubated for the indicated times, 10 μL (5 mg/mL) MTT was added to each well, and incubated at 37 °C for 4 h. Then, the supernatant was removed and dimethyl sulfoxide (DMSO) was added (150 μL/well). The optical density (OD) was detected at 570 nm with a model ELX800 Microplate Reader (Bio-Tek Instruments Inc., Winooski, VT, USA).

#### 3.3.2. Cell Morphologic Examination (HE Staining and Hoechst 33342 Staining)

The hematoxylin and eosin (H&E) stain assay kit was used for the morphology observation of HepG2 cells. Briefly, cells were fixed with 3% hydrogen peroxide for 10 min after washing with phosphate-buffered saline (PBS). The cells were then stained according to the manufacturer’s instructions and observed under a contrast inverted microscope (Olympus, Tokyo, Japan).

After 0, 24, and 48 h culturing, the cells were fixed with 4% paraformaldehyde for 10 min, and stained with 10 mg/mL of Hoechst 33342 for 10 min. The cells were washed twice by PBS for morphologic observation under fluorescence microscope.

#### 3.3.3. Flow Cytometry Analysis

After 0, 24, and 48 h culturing, cells were harvested and washed with cold PBS, and fixed by ethanol (70%, *v*/*v*). Then, cells were dissolved in 1 mL PBS containing RNase, PI, Triton X-100, and EDTA (pH 7.4), and incubated at 37 °C for 30 min. The apoptotic rate and the distribution of cells at different cell cycle stages was measured by BD FACSCallibur (BD Biosciences, Franklin Lakes, NJ, USA).

### 3.4. Statistical Analysis

The experiments were repeated three times. Statistical differences between groups were determined using one-way ANOVA. The level of *p* < 0.05 was considered to be statistically significant, and the level of *p* < 0.01 was considered to be statistically extremely significant.

## 4. Conclusions

The crude EPS was prepared from *S. thermophilus* CH9 grown in a modified skimmed milk-based medium, and then purified by DEAE-52 cellulose and SephadexG-100, respectively. Finally, three fractions, named EPS-1a, EPS-2a, and EPS-3a, were obtained for further research. We found that EPS-3a has the highest contents of protein and a special monosaccharide composition different from EPS-1a and EPS-2a. In addition, the surface morphology observed suggested that there were protein particles on the sugar chain of EPS-3a and EPS-3a was a protein-containing polysaccharide. Apart from this, we demonstrated that EPS-3a exhibited higher antitumor activity in vitro, which was associated with cell apoptosis as determined by typical morphological changes and flow cytometry analysis. This may provide useful ideas for the development of antitumor drugs. We suppose that the basic structure may be the key factor for activity, and further works are needed to investigate this.

## Figures and Tables

**Table 1:** Preliminary characterization of EPS-1a, EPS-2a, and EPS-3a.

Samples	Carbohydrate (%)	Protein (%)	Sugar Component (%)								
			Fuc	Rib	Rha	Ara	Xyl	Sor	Man	Glu	Gal
EPS-1a	96.32	-	-	-	-	-	-	30.18	69.82	-	-
EPS-2a	92.33	1.12	2.09	-	-	1.11	0.55	15.05	57.09	23.39	-
EPS-3a	85.62	2.52	1.07	0.63	0.59	0.35	0.41	2.03	-	63.93	36.07

**Table 2:** Effect of EPS-3a on cell cycle distribution of HepG2 Cells (*n* = 3).

Time	Apoptosis Cell (%)	G0/G1 (%)	S (%)	G2/M (%)
Control	2.59 ± 0.32	63.70 ± 0.45	14.90 ± 2.02	21.40 ± 0.94
24 h	7.45 ± 1.55 *	75.81 ± 0.33 *	19.10 ± 1.96	5.15 ± 1.86 *
48 h	14.70 ± 1.24 **	89.70 ± 2.01 **	5.33 ± 2.04 *	5.02 ± 1.84 **

Note: Compared with control, * *p* < 0.05, ** *p* < 0.01.
